# IRE1α deficiency promotes tumor cell death and eIF2α degradation through PERK dipendent autophagy

**DOI:** 10.1038/s41420-017-0002-9

**Published:** 2018-01-29

**Authors:** Antonello Storniolo, Vincenzo Alfano, Sabino Carbotta, Elisabetta Ferretti, Livia Di Renzo

**Affiliations:** 1 Department of Experimental Medicine, Sapienza University, 00161 Rome, Italy, Istituto di Ricovero e Cura a Carattere Scientifico Neuromed, Pozzilli 86077 Isernia, Italy; 2 Department of Molecular Medicine, Sapienza University of Rome, Rome, Italy; 3 Department of Surgical Sciences, Sapienza University of Rome, Rome, Italy; 4 Azienda Ospedaliera Universitaria Policlinico Umberto I, Rome, Italy

## Abstract

Sensors of endoplasmic reticulum (ER) stress function in a co-ordinated manner. In the present study we investigated the relationship between IRE1α and PERK pathways and survival of ER stressed U937 cells and BC3 cells. To this end, we investigated the effects of a subcytotoxic concentration of Tunicamycin in IRE1α-proficient and in IRE1α-deficient cells, by pharmacological inhibition with 4μ8 C or down-regulation by specific siRNA. We show that either type of IRE1α deficiency affects eIF2α expression and causes cell death increase. GSK2606414, a PERK inhibitor, and PERK specific siRNA prevent eIF2α down-regulation and restore cell survival. Degradation of this protein is due to autophagy, as it is prevented by bafilomycin and not by proteasome inhibition. Furthermore, activation of the autophagy flux is PERK dependent. Also the Cathepsin B inhibitor CA074 prevents eIF2α from degradation and reduces cell death. Altogether, these results show that IRE1α deficiency in ER stressed cells leads to an unexpected decrease of eIF2α, an important molecule for protein translation, through PERK dependent autophagy. Thus, IRE1/XBP1 inhibitors may represent a feasible strategy for tumor therapy, while PERK inhibitors may vanish the goal.

## Introduction

Most secreted and plasma membrane proteins are folded and matured in the endoplasmic reticulum (ER) lumen. Disturbances in ER calcium homeostasis and protein processing cause the accumulation of misfolded or unfolded proteins in the ER, a cellular condition referred to as “ER stress”. Adaptation to ER stress is mediated by the induction of the unfolded protein response (UPR), a regulated and complex signal transduction pathway transmitting information to the cytosol and nucleus to increase protein folding capacity of the ER^1–3^. The hallmark of the UPR is the upregulation of ER chaperones and folding enzymes, which are required to bind the unfolded proteins and prevent their aggregation^4^. Also a transient attenuation of protein synthesis participates to the UPR by limiting the load of proteins under conditions not well suited to their proper folding, while allowing the transcriptional upregulation of ER chaperones and folding enzymes^5^. However, cells undergo apoptosis when adaptation mechanisms are unable to alleviate the stress.^6,7^ Thus, the UPR serves to mitigate the stress, or, alternatively, to eliminate stressed cells in order to protect the organism.

Three resident ER transmembrane sensors detect unfolded proteins in the ER to initiate three distinct UPR branches: inositol-requiring protein-1α (IRE1α), activating transcription factor-6 (ATF6), and protein kinase RNA (PKR)-like ER kinase (PERK)^3–5,8^. IRE1α is an evolutionarily conserved from yeast to human dual enzyme, possessing both a Ser/Thr protein kinase and endoribonuclease activity. Upon BiP/GRP78 (immunoglobulin heavy chain binding protein/78 kDa glucose-regulated protein) dissociation, IRE1α dimerizes and autophosphorylates, thus, causing a conformational change that allosterically activates its endoribonuclease domain. Activated IRE1α, through its RNase domain, excises a 26 bp fragment from the mRNA encoding the transcription factor X-box-binding protein 1 (XBP1) in metazoans, by an unconventional splicing event that leads to generate XBP1s (“s” for spliced), a highly active transcription factor, a key regulator of ER folding capacity, controlling important genes involved in protein quality, ER translocation, glycosylation, and ER/Golgi biogenesis.^9,10^ XBP1 favors cell survival.^11^

PERK phosphorylates the eukaryotic translational initiation factor 2α (eIF2α), responsible of reducing protein synthesis and, therefore, the amount of proteins entering the ER.^12,13^ However, despite global translation inhibition, translation of ATF4 (Activating Transcription Factor 4) increases selectively, which upregulates the transcription factor C/EBP-homologous protein (CHOP)^14^. CHOP induction has been linked to apoptosis.^15,16^ It has been also observed that ATF4 and CHOP induce genes involved in autophagy^17^ and the growth arrest and DNA damage-inducible protein GADD34, a protein phosphatase (PP1) targeting protein that directs PP1 to dephosphorylate eIF2α^18,19^ and, therefore, to allow recovery from protein synthesis shutoff.^20^ It has been reported that PERK^-/-^ cells are hypersensitive to the lethal effects of ER stress.^21^ However, it is also known that silencing of PERK decreases apoptosis under saturated acid-induced cellular stress.^22^ And also, PERK silencing increases cell viability when ER stress is induced by silver nanoparticles and other data indicate that PERK silencing does not cause more cell death following ER stress.^23,24^ Thus, the role of PERK appears controversial.

Several data have indicated that either IRE1α or PERK-pathway play an important role in controlling autophagy-apoptosis crosstalk in ER stressed cells and that both pathways are necessary for the transcriptional upregulation of several autophagy genes.^25^ ER stress sensors function in a co-ordinated manner. IRE1α and PERK pathways are not independent each other, rather exists a regulatory connection between them. In the present study we set out to investigate the relationship between IRE1α and PERK pathways and death of ER stressed U937 leukemia cells and BC3 cells, derived from a pleural effusion lymphoma (PEL). To this end, we compared the effects of a subcytotoxic concentration of Tunicamycin (TN), an inhibitor of *N*-linked glycosylation, in IRE1α-proficient and IRE1α-deficient cells, by pharmacological inhibition or specific silencing.

## Results

### IRE1α plays a pro-survival role in cells under ER stress

TN caused death of U937 and BC3 cells in a dose-dependent manner (Supplementary Fig. 1a and 1b).^26^ In particular, TN 3 μM caused a slight increase of propidium iodide (PI) positive U937 cells (15 ± 6%) in comparison with untreated cultures (5 ± 1%), in addition to the appearance of 17 ± 8% subG1 events. Similarly, TN 3 μM caused in BC3 cells the appearance of 17 ± 4% PI positive cells and 16 ± 4% subG1 events, in comparison with 6 ± 1% PI positive cells and 7 ± 3% subG1 events in untreated cultures. SubG1 events were studied by cytofluorimetry of cell cycle phases of cells fixed and then stained with PI. The events preceding the narrow peak of G1 events, and therefore with a hypodiploid content of DNA, are considered to be indicative of apoptotic nuclei.^27^ These results suggest that TN 3 μM causes activation of a pro-survival pathway in U937 or BC3 cells. Therefore, this dose was used through the experiments here performed and cell death parameters were investigated after 18 h treatments.

**Fig. 1:**
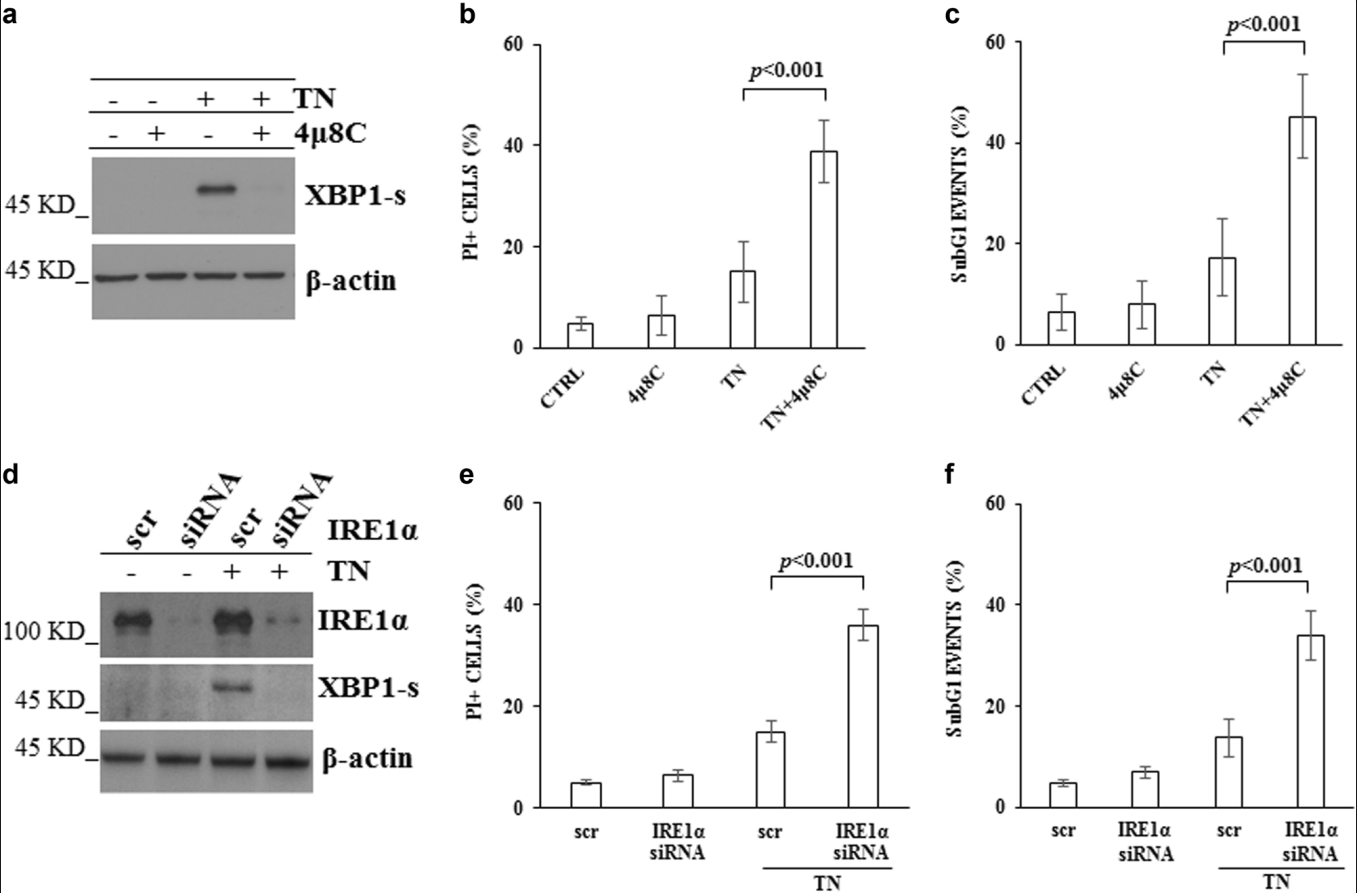
Pharmacological inhibition of IRE1α with 4μ8 C or IRE1α specific siRNA affect survival of U937 cells under ER stress **a** Western blot detection of XBP1-s in the lysates of U937 cells treated or not for 18 h with 4μ8 C (12.5 μM), TN (3 μM) or both drugs. Blotted proteins were probed with anti XBP1-s, followed by peroxidase-conjugated secondary antibody. The level of β-actin is shown at the bottom as a loading control. Representative blot out of three experiments is shown. **b, c** Cell death parameters were evaluated in U937 cells, treated as above indicated, by calculating PI positive cells as percentage of total cells examined by cytofluorimetry **b**. A portion of these cells were fixed and stained with PI and subG1 events were evaluated in the cell cycle by cytofluorimetry **c**. ≥10.000 events were acquired for each sample. In comparison with TN treatment alone, pretreatment with 4μ8 C caused a significant increase of cell death and statistical analysis by Student’s *t*-test are shown (*N* = 8). **d** Western blot detection of IRE1α and XBP1-s in the lysates of U937 cells transfected with equal amount of scr-siRNA or IRE1α specific siRNA for 72 h and, thereafter, treated or not with TN (3 μM) for further 18 h. Blotted proteins were probed with antibodies anti IRE1α or anti XBP1-s, followed by peroxidase-conjugated secondary antibody. The level of β-actin is shown at the bottom as a loading control and also to confirm the specificity of the transfected siRNA. Representative blots out of three experiments are shown. **e,f** Cell death parameters were evaluated in U937 cells transfected with equal amount of scr-siRNA or IRE1α specific siRNA for 72 h and, therafter, treated or not with TN (3 μM) for further 18 h. **e** PI positive cells were evaluated as percentage of total cells examined by cytofluorimetry. **f** subG1 events were evaluated in the cell cycle by cytofluorimetry. ≥10.000 events were acquired for each sample. Statistical analysis by Student’s *t* test are shown (*N* = 3)

As TN activates ER stress sensors, we set out to identify IRE1α role in TN activated survival pathway. Therefore, IRE1α activation was confirmed by XBP1-s detection in western blotting in U937 cell lysate (Fig. 1a). To be noted, XBP1-s was still present after 18 h of ER stress, suggesting a cellular need for this protein, whereas IRE1α pharmacological inhibition with 4μ8C^28^ prevented its expression (Fig. 1a) and caused a significant increase of PI positive cells (39 ± 6%) and subG1 events (45 ± 8%) in TN treated U937 cells (Fig. 1b, c). To further validate the above results, U937 cells were transfected with scr-siRNA or IRE1α-siRNA for 72 h, followed by 18 h treatment with TN 3 μM or none. Knockdown efficiency of IRE1α was about 85%, still effective after TN cell treatment, and able to prevent XBP1-s expression (Fig. 1d). Furthermore, specific IRE1α silencing was not cytotoxic per se, whereas it increased cell death after TN treatment (Fig. 1e, f). Similar results were obtained in BC3 cells (Supplementary Fig. 1c and d).

### eIF2α expression is down-regulated in IRE1α deficient and ER stressed cells

**Fig. 2:**
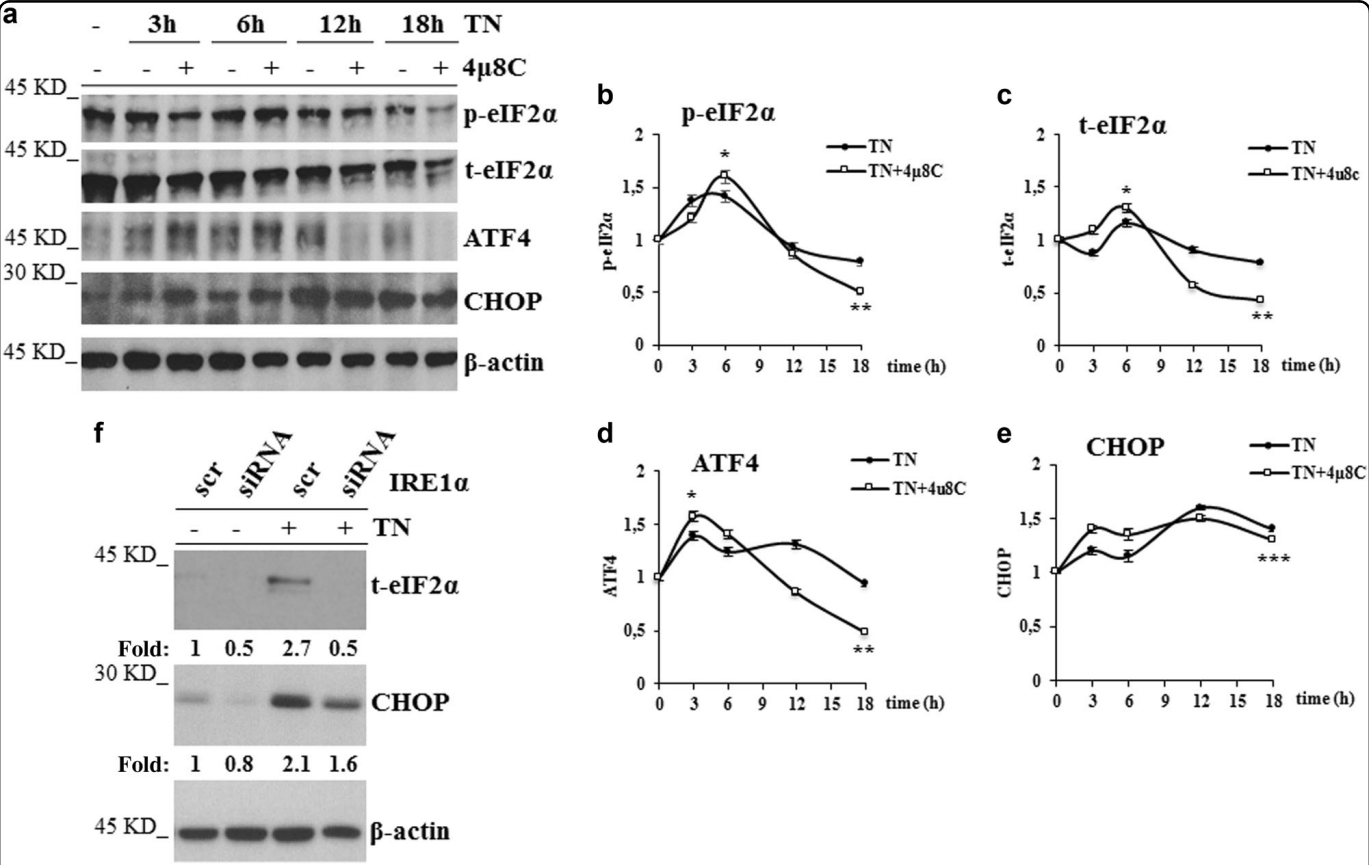
IRE1α impairment affects eIF2α expression **a** Representative western blots out of three experiments are shown for p-eIF2α, t-eIF2α, ATF4 and CHOP detected in the lysates of U937 cells treated for 18 h with none, TN (3 μM) or 4μ8 C (12.5 μM) + TN (3 μM) for the indicated hours. Each protein was probed with specific antibody followed by peroxidase-conjugated secondary antibodies. β-actin is shown as loading control. **b, c, d, e** Quantitative analysis of each protein was performed by densitometry of the relative bands in 3 experiments. Values were obtained using the following formula: (densitometry value of the band under examination / densitometry value of the band of the corresponding β-actin)/(densitometry value of the band under examination in the control / densitometry value of the β−actin band in the control). Values are means ± S.D. The statistical differences (Student’s *t*-test) between the values obtained in 4μ8 C + TN cell treatments v/s the values obtained in the corresponding TN cell treatments are indicated as: *P < 0.05, **P < 0.01, ***P > 0.05. **f** IRE1α impairment through specific silencing affects eIF2α expression. U937 treated for 72 h with equal amount of scrambled siRNA or IRE1α specific siRNA were treated or not with TN (3 μM) for 18 h. t-eIF2α and CHOP were analyzed by western blot in the cell lysates by specific antibodies followed by peroxidase-conjugated antibodies. β-actin is shown as loading control and also to confirm the specificity of the transfected siRNA. Values under each band were obtained using the following formula: (densitometry value of the band under examination / densitometry value of the band of the corresponding β-actin) / (densitometry value of the band of the protein under examination in the lysate of scrambled treated cells / densitometry value of the β-actin band in the lysate of scrambled treated cells)

The above results suggest that TN activates IRE1α playing a pro-survival role and that, upon its impairment, ER stress may be sensed by the other sensors in a pro-death manner. To explore the mechanism causing cell death in IRE1α deficient cells, we examined in TN and in 4μ8 C + TN treated U937 cells the expression of proteins of PERK pathway. Thus, p-eIF2α, t-eIF2α, ATF4 and CHOP were analyzed in western blot (Fig. 2a). Densitometric quantification of bands obtained in repeated experiments showed that, in comparison with untreated cells, TN led to a transient increase after 3–6 h of p-eIF2α (1.4 fold), after 6 h of t-eIF2α (1.2 fold), after 3 h of ATF4 (1.3 fold) and caused after 3–6 h a slight increase of CHOP, more evident after 18 h, when p-eIF2α, t-eIF2α and ATF4 declined to their baseline level (Fig. 2b–e). In comparison with untreated cells, TN together with IRE1α inhibitor led to a transient larger increase after 6 h of p-eIF2α (1.6 fold) and of t-eIF2α (1.4 fold), and after 3 h of ATF4 (1.6 fold). This combined treatment led after 3 h to an increase also of CHOP (1.4 fold). Interestingly, after 12–18 h, the levels of t-eIF2α and ATF4 became lower (0.5 fold) than in control cells (Fig. 2c, d), while CHOP remained elevated (Fig. 2e). Thus, these results show that either TN or 4μ8 C + TN treatments cause an initial (within 3–6 h) activation of PERK pathway, followed after 12–18 h by its decline. However, in comparison with TN treatment, the combined treatment led to an initial significant (*p* < 0.05) increase of t-eIF2α, followed by a significant (*p* < 0.01) decrease, below its constitutive level. ATF4 expression depends on p-eIF2α and, as expected, paralleled that. Of note, either TN or the combined treatment with 4μ8 C + TN caused similar CHOP persistence over time. Similar results were obtained with BC3 cells: in comparison with untreated cells, the combined treatment with 4μ8 C + TN led after 18 h to eIF2α decrease (Supplementary Fig. 2a). In order to further validate these results, t-eIF2α and CHOP were analyzed by western blot in U937 cells transfected with scr-siRNA or IRE1α-siRNA for 72 h, followed by 18 h TN treatment or none: in scr-siRNA treated cells t-eIF2α was well represented and increased 2.1 fold after TN treatment, whereas it appeared already down-regulated in U937 cells transfected with IRE1α−siRNA, even before TN treatment (0.4 and 0.4 fold, respectively). CHOP analysis in the same cell lysates showed that the level of this protein was similarly increased by TN in either scrambled or IRE1α-siRNA treated cells (1.9 and 1.7 fold, respectively) (Fig. 2f).

Thus, IRE1α impairment in TN stressed cells modifies PERK pathway and, moreover, leads to a decreased expression of eIF2α.

### PERK is responsible of eIF2α down-regulation and of the increased death in IRE1α deficient cells, under ER stress

**Fig. 3:**
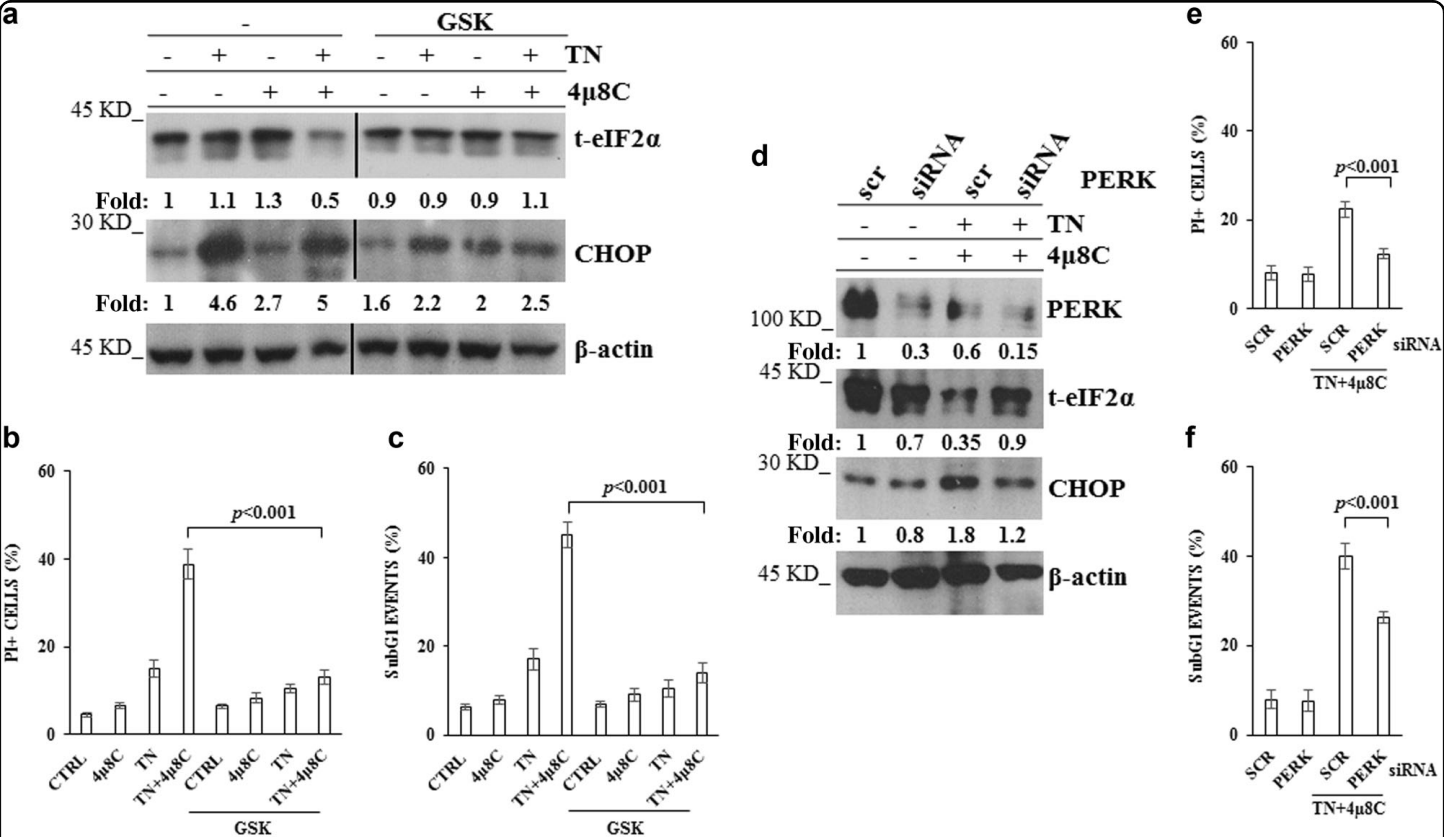
PERK impairment prevents eIF2α down-regulation and cell death **a** Representative western blot out of three experiments for t-eIF2α and CHOP detected in the lysates of U937 cells pretreated or not for 30 min with GSK (10 μM) and thereafter treated or not for 18 h with TN (3 μM), 4μ8 C (12.5 μM) or both drugs. Each protein was probed with specific antibody followed by peroxidase-conjugated secondary antibodies. β-actin is shown as loading control. The values under each band were obtained using the following formula: (densitometry value of the band under examination / densitometry value of the band of the corresponding β-actin) / (densitometry value of the band under examination in the lysate of untreated cells / the densitometry value of the β-actin band in the lysate of untreated cells). **b, c** Cell death parameters were evaluated in U937 cells, treated as in **a**, by calculating PI positive cells as percentage of total cells examined by cytofluorimetry **b** and subG1 as percentage of the events in the cell cycle examined by cytofluorimetry **c**. For each parameter ≥ 10.000 events were acquired for each sample. The reported values are the means ± S.D. (*N* = 5). Statistical analysis by Student’s *t*-test are shown. **d** PERK impairment by specific siRNA restores eIF2α expression. U937 treated for 72 h with equal amount of scrambled siRNA or PERK specific siRNA were treated or not with 4μ8 C (12.5 μM) + TN (3 μM) for 18 h. PERK, t-eIF2α and CHOP were analyzed by western blot in the cell lysates by specific antibodies followed by peroxidase-conjugated antibodies. β-actin is shown as loading control and to confirm the specificity of the transfected siRNA. The values under each band were obtained using the formula: (densitometry value of the band under investigation / densitometry value of the band of the corresponding β-actin) / (densitometry value of the band under investigation in the lysate of scrambled treated cells / the densitometry value of the β-actin band in the lysate of scrambled treated cells). This type of experiment was performed twice with comparable results. **e, f** Death parameters were evaluated in U937 cells, treated as in **d**, by calculating PI positive cells as percentage of total cells examined by cytofluorimetry **e** and subG1 as percentage of the events in the cell cycle examined by cytofluorimetry **f**. For each parameter ≥ 10.000 events were acquired for each sample. The reported values are the means ± S.D. (*N* = 3). Statistical analysis by Student’s *t*-test are shown

The above results let us investigate PERK role in the regulation of eIF2α expression and in the fate of IRE1α deficient cells, under TN treatment. Thus, U937 cells were pretreated with GSK2606414 (GSK), a PERK inhibitor^29,30^, and thereafter treated or not with TN, 4μ8 C, or 4μ8 C + TN. Interestingly, GSK reverted the above results: it blocked t-eIF2α decrease in IRE1α deficient U937 cells and it prevented by about 50% CHOP expression in either IRE1α-deficient or proficient cells (Fig. 3a). Therefore, we examined the role of PERK in deciding the fate of IRE1α-deficient U937 cells, under TN treatment. To this end, cells were pretreated with GSK and thereafter treated with 4μ8 C, TN or 4μ8 C + TN. GSK prevented TN and, moreover, 4μ8 C + TN induced cell death (Fig. 3b, c). Similar results were obtained in BC3 cells pretreated with GSK and thereafter treated with 4μ8 C, TN or 4μ8 C + TN (Supplementary Figs. 2A, B and C). Actually, in these cells we observed also an increased expression of t-eIF2α upon pharmacological inhibition of IRE1α (Supplementary Fig. 2A). To further validate these results PERK expression was silenced in U937 cells by specific siRNA: in comparison with scr-siRNA treated cells, specific silencing caused a decrease of about 70% of PERK, still evident in TN + 4μ8 C treated cells (Fig. 3d). Furthermore, PERK-siRNA prevented 4μ8 C + TN induced t-eIF2α decrease and CHOP increase (Fig. 3d). Similarly to PERK pharmacological inhibition, also knockdown of this sensor prevented 4μ8 C + TN induced cell death (Fig. 3e, f). These results confirm a cross-talk between IRE1α and PERK in ER stressed cells and they show that eIF2α undergoes an unexpected PERK dependent decrease in IRE1α-deficient cells, that may be relevant for cell survival, being eIF2α a key molecule at the crossroad of multiple pathways activated in stressed cells, in order to restore cellular homeostasis. Thus, in ER stressed cells, IRE1α impairment leads to t-eIF2α decrease and increased cytotoxicity, PERK mediated.

### eIF2α is degraded through PERK dependent autophagy

**Fig. 4:**
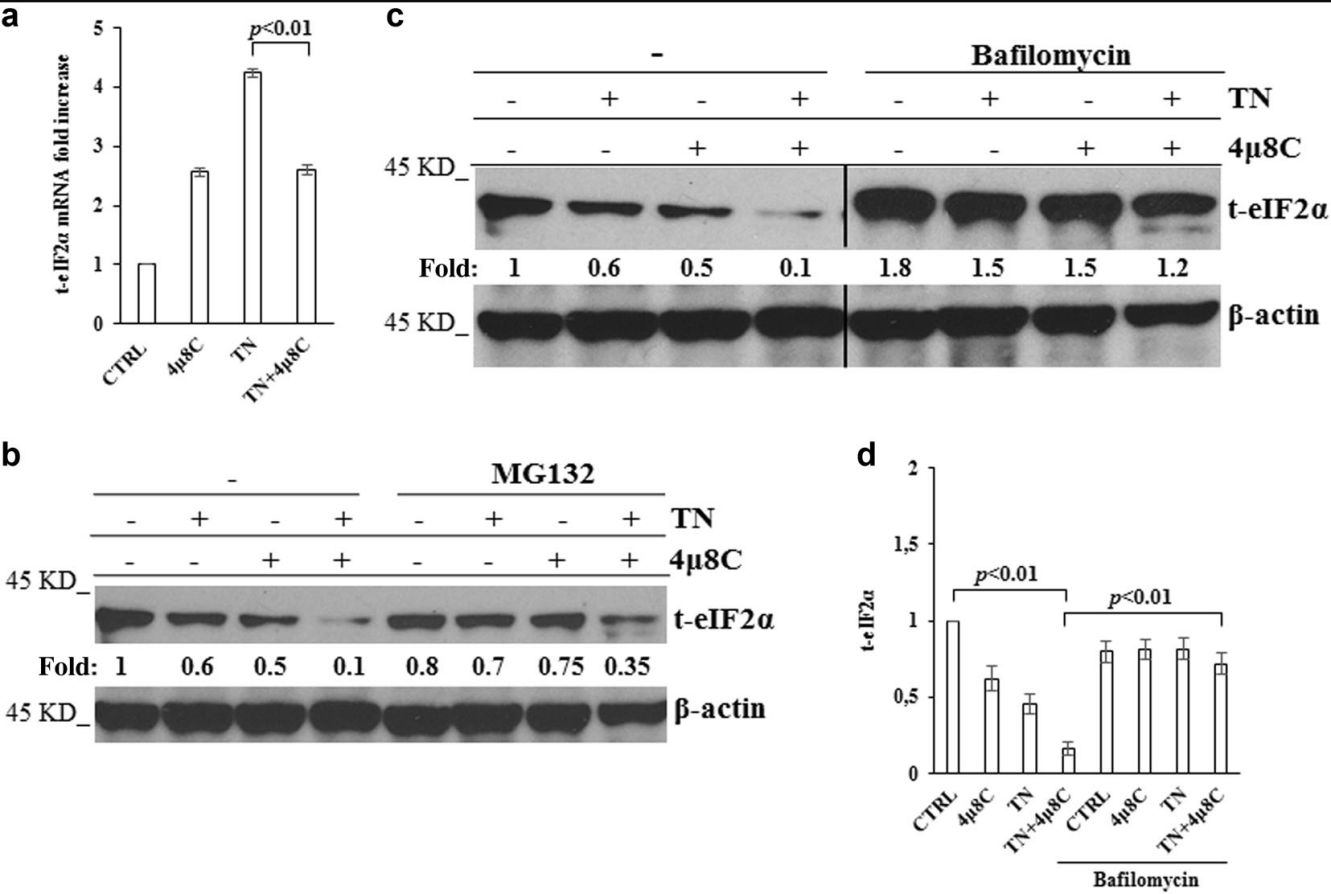
eIF2α expression is restored by bafilomycin and not by proteasome inhibitor **a** Quantitative RT-PCR of eIF2α mRNA in U937 cells treated or not for 18 h with 4μ8 C (12.5 μM), TN (3 μM) or both drugs. The reported values are means ± S.D. (*N* = 3). Statistical analysis by Student’s *t*-test is shown. **b, c** Representative western blots out of three experiments are shown for t-eIF2α detected in the lysates of U937 cells treated or not with TN (3 μM), 4μ8 C (12.5 μM) or both drugs for 18 h and in the last 6 h treated or not with MG132 (50 μM) or with Bafilomycin (50 nM). β-actin is shown as loading control. The values under each band were obtained using the formula: (densitometry value of the band under investigation / densitometry value of the band of the corresponding β-actin) / (densitometry value of the band under investigation in the lysate of untreated cells / the densitometry value of the β-actin band in the lysate of untreated cells). **d** Quantitative analysis of total eIF2α detected in the western blots of three independent experiments was performed as above indicated. Results are expressed as mean ± S.D. and statistical analysis by Student’s *t*-test are shown

**Fig. 5:**
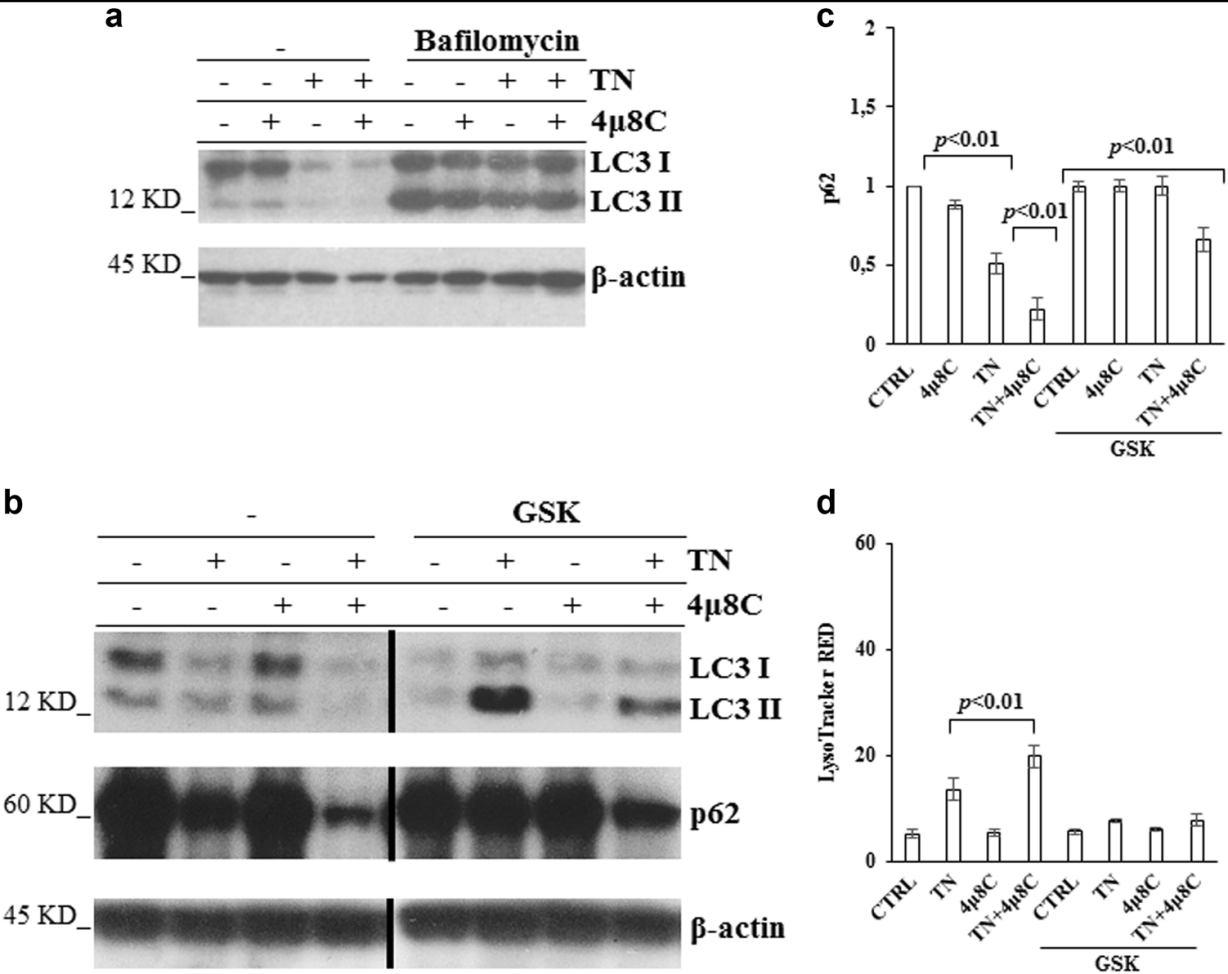
IRE1α deficiency causes increased autophagy, PERK dependent **a** Representative western blot out of three experiments is shown for LC3I-II detected in the lysates of U937 cells treated or not with 4μ8 C (12.5 μM), TN (3 μM), or both drugs for 18 h and in the last 3 h treated or not with bafilomycin (50 nM). β-actin is shown as loading control. Specific primary antibodies and peroxidase-conjugated antibodies were used. **b** Representative western blots for LC3I-II and p62 detected in the lysates of U937 cells pretreated or not with GSK (10 μM) for 30 min and thereafter treated or not for 18 h with TN (3 μM), 4μ8 C (12.5 μM) or both drugs. LC3I-II and p62 were probed with specific antibodies followed by peroxidase-conjugated secondary antibodies. β-actin is shown as loading control. **c** Quantitative analysis of p62 was performed by densitometry of the relative bands in the western blots of three experiments. Each value was obtained using the following formula: (densitometry value of the band under examination / densitometry value of the band of the corresponding β-actin)/(densitometry value of the band under examination in the control / densitometry value of the β−actin band in the control). The values are means ± S.D. Statistical differences (Student’s *t*-test) are indicated. **d** Cells were pretreated or not with GSK (10 μM) and then treated or not for 18 h with TN (3 μM), 4μ8 C (12.5 μM) or both drugs. Thereafter, cells were stained with LysoTracker Red and analyzed by flow cytometry. The Mean Fluorescence Intensities were measured from the histograms representing FL3(log) in ≥ 10.000 events. Data are means ± S.D. of three independent experiments. Statistical analysis by Student’s *t*-test is shown

Quantitative RT-PCR showed that, in comparison with U937 untreated cells, TN induced a 4 fold increase of eIF2α transcript, that was prevented in a significant manner (*p* < 0.01) by 4μ8 C, although the inhibitor per se caused an increase of the transcript (Fig. 4a). Therefore, eIF2α decrease in 4μ8 C + TN treated cells depends on transcriptional and post-transcriptional events. Proteasome and autophagy are protein degradative mechanisms. Thus, we inhibited the proteasome with MG132 or autophagy with bafilomycin and analyzed t-eIF2α by western blot in the lysates of untreated, 4μ8 C, TN or 4μ8 C + TN treated cells. MG132 did not restore t-eIF2α level in 4μ8 C + TN treated cells, indicating that this protein was not degraded through this route (Fig. 4b). Then, U937 cells were treated or not with 4μ8 C, TN or 4μ8 C + TN for 18 h and in the last 6 h were treated or not with bafilomycin to block autophagosomes degradation.^31^ Interestingly, t-eIF2α levels were restored in bafilomycin treated cells (Fig. 4c, d). These results suggested autophagy involvement in eIF2α degradation. Thus, we analyzed the autophagy flux and detected that 4μ8 C causes an increase of TN induced flux. In fact, TN caused in 4μ8 C treated cells a large increase of LC3-II after block of autophagy degradative steps by bafilomycin (Fig. 5a) and a large decrease of p62 (Fig. 5b, c). U937 cells were also stained with LysoTracker-Red DND99, a fluorescent probe selective for acidic organelles. LysoTracker-Red fluorescence was more pronounced in TN treated cells (MFI = 13 ± 2) in comparison with untreated or 4μ8 C treated cells (MFI = 5.5 ± 0.5 and 5.6 ± 0.5, respectively), while 4μ8 C + TN caused the largest accumulation of the dye (MFI = 20 ± 2) (Fig. 5d). These results suggest that eIF2α is actively degraded through the autophagy flux in ER stressed and IRE1α-deficient cells.

To explore PERK role in the autophagy flux causing eIF2α degradation, western blot analysis of LC3I/II and p62 was performed in the lysates of U937 cells pretreated with GSK and thereafter subjected to ER stress with TN or 4μ8 C + TN. This approach showed that GSK caused LC3-II accumulation and prevented p62 decrease, indicating that PERK was involved in the regulation of the autophagy flux occurring in TN and in 4μ8 C + TN treated cells (Fig. 5b, c). Interestingly, GSK pretreatment per se did not modify the fluorescence of untreated cells (MFI = 5.6 ± 0.5) and of 4μ8 C treated cells (MFI = 5.6 ± 1), while it did not allow the fluorescence development caused by TN (MFI = 6 ± 0.5) or by 4μ8 C + TN (MFI = 6.2 ± 1) (Fig. 5d). Similar results were obtained also with BC3 cells, as GSK caused an increased p62 expression (Supplementary Figs. 3A and B) and prevented the LysoTracker-Red fluorescence in 4μ8 C + TN treated cells (Supplementary Fig. 3C). Thus, IRE1α impairment causes PERK dependent activation of autophagy leading to eIF2α degradation.

### CA074, a cathepsin B inhibitor, prevents eIF2α degradation and cell death

**Fig. 6:**
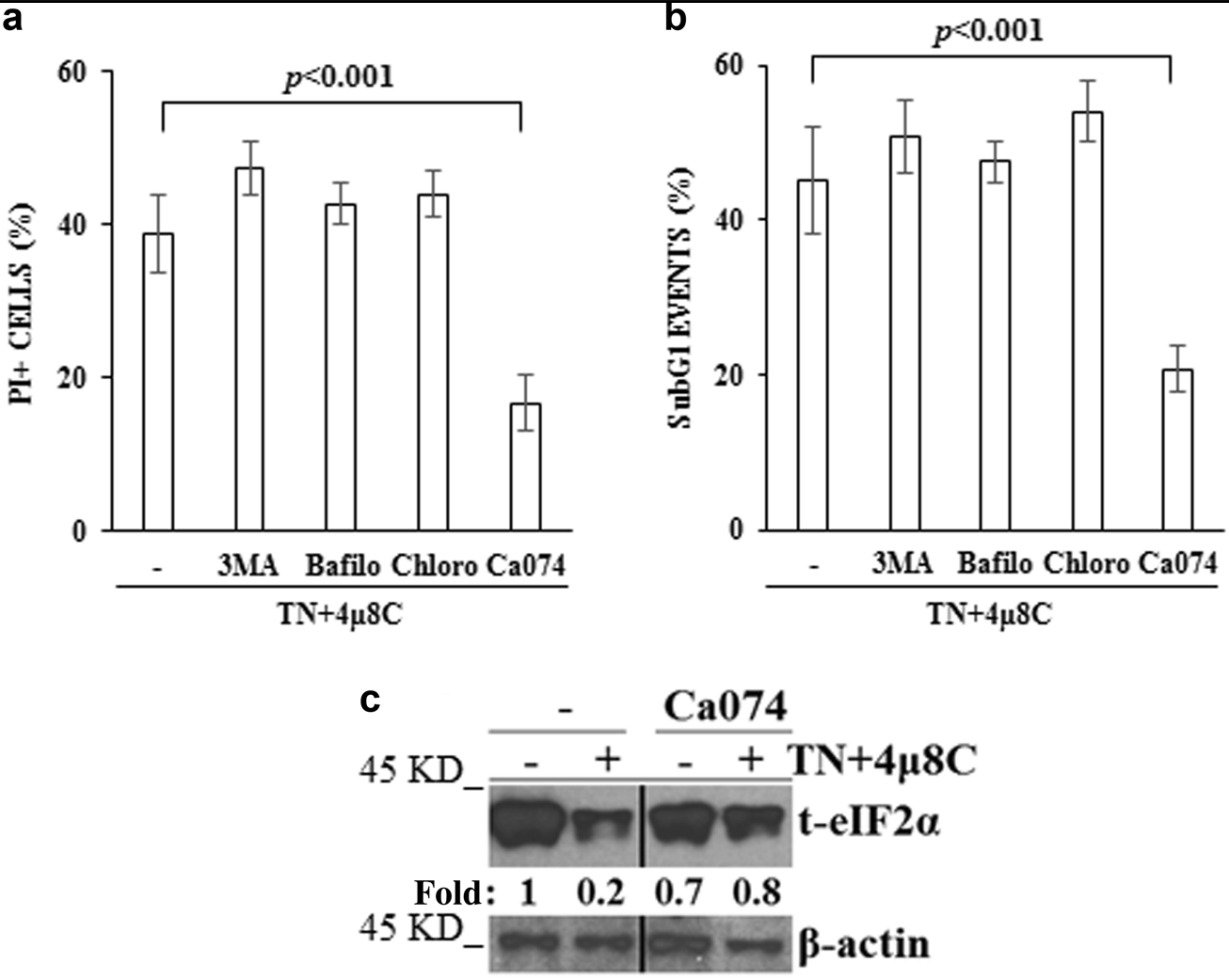
CA074 prevents eIF2α degradation and cell death **a,b** U937 cells were treated with 4μ8 C (12.5 μM) + TN (3 μM) for 18 h and in the last 6 h also with or without 3-MA (5 mM), Bafilomycin (50 nM), Chloroquine (200 μM) or CA074 (10 μM). Cell death parameters were evaluated by calculating PI positive cells as percentage of total cells examined by cytofluorimetry **a**. A portion of these cells were fixed and stained with PI and subG1 events were evaluated in the cell cycle by cytofluorimetry **b**. For each parameter ≥ 10.000 events were acquired for each sample. The reported values are means ± S.D. (*N* = 3). Statistical analysis by Student’s *t*-test are shown. **c** Representative western blot for eIF2α detected in the lysates of U937 cells treated or not with 4μ8 C (12.5 μM) + TN (3 μM) for 18 h and with or without CA074 during the last 6 h. β-actin is shown as loading control. Specific primary antibodies and peroxidase-conjugated antibodies were used. The values under each band were obtained using the following formula: (densitometry value of the band under investigation / densitometry value of the band of the corresponding β-actin) / (densitometry value of the band under investigation in the lysate of untreated cells / the densitometry value of the β-actin band in the lysate of untreated cells). This experiment was repeated twice with comparable results

If eIF2α degradation was responsible of cell demise following ER stress in IRE1α-deficient cells, inhibition of autophagy should prevent its degradation and, therefore, cell death. However, either 3-MA or bafilomycin or chloroquine addition in the last 6 h of 18 h cell treatment with TN + 4μ8 C did not prevent cell death induced by these drugs (Figs. 6a, b). However, TN-induced ER stress causes disfolded/aggregated proteins and cells need autophagy to degrade them. Therefore, we used lysosome enzymes inhibitors and detected that CA074, a Cathepsin B inhibitor, added during the last 6 h of 18 h treatment with TN + 4μ8 C, prevented cell death (Figs. 6a, b) and, interestingly, prevented eIF2α decrease in 4μ8 C + TN treated cells (Fig. 6c).

These results indicate that, in cells under ER stress, IRE1α deficiency promotes activation of PERK pathway, activating autophagy and eIF2α degradation through the proteolytic activity of lysosome enzymes and this mechanism is responsible of cell death.

## Discussion

In the present study we show for the first time that eIF2α, an important molecule for protein translation, is degraded through PERK dependent autophagy in IRE1α-deficient and ER stressed cells.

IRE1α deficiency was obtained by specific silencing or the pharmacological inhibitor 4μ8 C. In both cases, TN promoted an increased cell death and modified the activation of PERK pathway. The investigated proteins belonging to this pathway are eIF2α, ATF4 and CHOP. Unphosphorylated eIF2α allows protein synthesis, while its phosphorylated form, not allowing protein synthesis, alleviates ER stress.^32^ ATF4 is an important transcription factor, promoting cell survival through activation of genes involved in aminoacid metabolism, redox reactions and protein secretion.^33^ CHOP is a transcription factor, playing a pro-apoptosis role.^15,16^

The activation of PERK pathway here observed can be examined at early and late times, i.e. at 3–6 h and at 18 h of cell treatment with TN with or without the pharmacological inhibitor 4μ8 C. Indeed, in comparison with IRE1α-proficient cells, eIF2α and ATF4 appeared increased in the first 3–6 h of IRE1α inhibition, while they decreased thereafter, below their respective constitutive levels, whereas CHOP expression was only slightly affected in IRE1α–deficient cells. The late results were obtained also after IRE1α silencing, thus, rouling out possible off-target effects of the inhibitor.

ATF4 down-regulation in IRE1α-deficient cells paralleled that of t-eIF2α, thus, supporting that its expression during ER stress is linked to eIF2α. Also CHOP could be expected to be strongly down–regulated, being its translation under ATF4 control. However, this was not the case and could depend on the half-life of this protein, but also on the fact that CHOP may be transcribed under the activity of ATF6, another sensor of ER stress.^34^ However, the similar expression of CHOP in IRE1α-proficient and -deficient cells suggests that it is not responsible of the increased cell death observed in IRE1α deficient cells.

These results indicate the existence of a molecular cross-talk between ER stress sensors, where IRE1α controls PERK pathway. Future studies will explain how IRE1α controls PERK and its pathway.

In this study we investigated the molecular terms leading to eIF2α down-regulation and found that it depended on PERK, as inhibition or specific siRNA of this sensor prevented eIF2α decline in IRE1α-deficient cells. In the same cells CHOP was only in part increased, and cell death was prevented. These results confirm the link between PERK and CHOP expression and suggest a new mechanism linking PERK and eIF2α, as this protein, in IRE1α absence, would be down-regulated under PERK control.

Protein decrease can be due to decreased transcription and/or increased degradation. For what concerns eIF2α, we observed a partial decrease of its mRNA in cells treated with 4μ8 C + TN, in comparison with cells treated only with TN. This finding suggests that IRE1α contributes to the regulation of the transcription of *eIF2*α, without excluding not transcriptional mechanisms. Thus, we examined cell machineries degrading proteins. The proteasome was inhibited with MG132, while the autophagy flux was blocked with bafilomycin: eIF2α expression was restored to normal levels only upon autophagy inhibition. Of interest, in comparison with TN treatment, 4μ8 C + TN caused a more conspicous increase of the acidic compartment, as shown by the LysoTracker Red fluorescence, a larger decrease of p62, known to be degraded in the final steps of autophagy, and an increased degradation of LC3-II, as indicated by bafilomycin experiments. Thus, all together, IRE1α deficiency leads ER stressed cells to eIF2α degradation through a more sustained autophagy flux.

It is known that IRE1α/XBP1 deficiency leads to augmented autophagy, enhancing the clearance of the mutant superoxide dismutase-1 (SOD1) protein.^35^ However, in those studies the relationship between IRE1α and PERK was not investigated, while our results indicate a link between IRE1α deficiency and PERK dependent activation of autophagy.

It is known that eIF2α and ATF4, under PERK activation, are responsible of *LC3* transcription and autophagy activation.^36^ And, indeed, we observed that either TN or TN + 4μ8 C activate autophagy through PERK involvement. In fact, GSK prevented the decrease of LC3-II and of p62 following TN + 4μ8 C cell treatment. These findings show PERK involvement in autophagy regulation and, therefore, in eIF2α degradation and they suggest a relationship between intensity of PERK-pathway activation and intensity of autophagy activation, even if the increase of eIF2α and ATF4 occurring in the initial 6 h of ER stress should be responsible of the increased autophagy flux still occurring after 12–18 h. However, it would be also possible that PERK enters into autophagy activation through different and, eventually, selective mechanisms, without the involvement of eIF2α/ATF4 pathway.

Autophagy is responsible of degradation of proteins and organelles by lysosome enzymes in order to maintain cellular omeostatic conditions. In this way, cells under ER stress degrade aggregated proteins and survive. However, conditions causing too long or excessive autophagy may lead to cell death.^37,38^ Here we observed that in ER stressed and IRE1α-deficient cells inhibition of autophagy by 3-MA or chloroquine or bafilomycin was slightly more cytotoxic. Interestingly, CA074 did not allow eIF2α degradation and increased cell survival. This inhibitor blocks a portion of lysosome enzymes, like Cathepsin B, leaving other enzymes still efficient in degrading the autophagosome cargo. Thus, the cells would survive, as they benefit of the ongoing autophagy flux and the presence of eIF2α.

In aminoacid starvation, proteins involved in translation, such as ribosomal proteins and translation initiation factors, undergo a pronounced degradation.^39^ Ribosomes appear to be stable within 5 h of starvation and to be degraded specifically by autophagy in long term starvation. Thus, it would be possible that also eIF2α undergoes degradation through autophagy at a late time, i.e. around 12–18 h, of ER stress in IRE1α deficient cells. Whether this condition would resemble amino-acid starvation or represents a new condition of autophagy activation remains to be investigated.

Altogether, the results here reported reinforce the possibility that IRE1α/XBP1 inhibitors, by causing eIF2α degradation, may represent a strategy for tumor treatment and, moreover, these results indicate that the increased autophagy is PERK dependent and, therefore, that conditions affecting this sensor may vanish the proposed goal.

## Materials and methods

### Materials

Antibody anti-SQSTM1/p62 was from BD Transduction Laboratories (San Jose, CA, USA). Antibodies anti-BiP, -CHOP, phoshorylated-eIF2α (p-eIF2α), total–eIF2α (t-eIF2α), -IRE1α, -LC3I/II and horseradish peroxidase (HRP)-conjugated anti-rabbit- and anti-mouse-immunoglobulin antibodies were from Cell Signaling Technology (Danvers, MA, USA). Antibody anti-β actin, bafilomycin, bovine serum albumin (BSA), chloroquine, HBSS, l-glutamine, 3-methyladenine, penicillin-streptomycin, phosphate buffered saline (PBS), PI, RNAse, and RPMI-1640 were from Sigma-Aldrich (St. Louis, MO, USA). Fetal calf serum (FCS) (GIBCO), LysoTracker-Red, Lipofectamine RNAiMAX and OPTI-MEM medium (GIBCO), siRNA-IRE1 (Ambion), siRNA-PERK (Ambion) and scrambled siRNA (Ambion) were from Life Technologies (Invitrogen, San Giuliano Milanese, Italy). RC-DC protein assay, SDS-sample buffer, protein standard, SDS-PAGE reagents and polyvinylidene difluoride membranes were from Bio-Rad Laboratories (Segrate, Italy). ECL fast femto was from Società Italiana Chimici (Rome, Italy). TN, 4μ8c and GSK2606414 were from Calbiochem (San Diego, CA, USA).

### Cells and cell viability

U937 cells, a human monoblastic leukemia cell line, and BC3 cells, a human B-cell line derived from a PEL carrying latent Kaposi’s Sarcoma-associated Herpes Virus (KSHV), were grown in RPMI-1640 medium supplemented with 5% heat-inactivated FCS, 2 mM glutamine, 100 units/ml penicillin and 100 μg/ml streptomycin, at 37°C, in fully humidified 95% room air/5% CO_2_. Cells were resuspended three times a week in fresh complete medium to 3 × 10^5^/ml.Cells in every experiment were ≥94% viable, as assessed by calculating alive trypan blue-excluding cells as percentage of all cells counted. They were washed, resuspended in complete medium, 1 × 10^6^/ml, transferred to multi-well plates and then treated with inhibitors or vehicles, incubated for 30 min, and subsequently exposed to test agents or, again, to vehicles. At the end of each experiment, cells were gently mixed and aliquots taken for PI staining and cell cycle analysis. Vehicles, even when used in combination, were ≤0.3% (v/v) and did not modify any investigated parameter in comparison with control cultures.

### Flow cytometry analysis of cell death

Nuclear DNA fragmentation was quantified by flow cytometry evaluation of hypodiploic (subG1) DNA events after cell fixation and PI staining.^27,40^ Briefly, cells were washed with PBS, pelletted and fixed in ice cold ethanol/water (70/30, v/v) for 1 h, pelletted again, washed twice with PBS, and finally resuspended in PBS containing RNAse (20 μg/ml) and PI (100 μg/ml). Events in the different cell cycle phases were gated manually using an EPICS XL cytofluorimeter (Beckman Coulter, Hialeah, Fl, USA). At least 10.000 events/sample were acquired. Collected data were analyzed using the Multicycle software for DNA content and cell cycle analysis (Phoenix Flow System, San Diego, CA, USA). SubG1 events, representative of apoptotic cells, are given as percentage of the total cell population.

Membrane permeability, indicative of cell death, was investigated by resuspending the cells in HBSS containing PI (20 μg/ml) at room temperature and analyzed by flow cytometry (EPICS-XL), measuring the fluorescence emission at >575 nm (FL3log).

### Western blot analysis

Whole cell lysates were prepared as previously described.^26^ Briefly, the cells were kept for 30 min on ice in lysis buffer (NaCl 150 mM, CaCl2 1 mM, MgCl2 1 mM, NaN3 0.1%, NaF 10 mM, Triton X-100 1% (v/v), orthovanadate 1 mM, aprotinin 2 μg/ml, leupeptin 2 μg/ml, iodoacetamide 10 mM, PMSF 2 mM, and pepstatin 20 μM). Appropriate volumes of 4x SDS-sample buffer (v/v) were then added. Cell lysates were briefly sonicated, warmed at 95°C for 5 min, and cleared by 14.000 x g centrifugation in a microfuge for 15 min at 4°C. Supernatants were collected and proteins were quantified by RC-DC protein assay. Equal protein amounts were separated from the different samples by SDS-PAGE and blotted onto PVDF membranes. Transfer efficiency was checked with Ponceau staining. Blots, blocked in tris-buffered saline containing BSA 5% (w/v), were probed with specific primary antibodies, washed with PBS-Tween-20, and then incubated with peroxidase-conjugated secondary antibody. Finally, each membrane was probed to detect β-actin. The final dilutions and incubation times suggested by the manufacturer were used for each antibody. Immunodetection was performed using the ECL reagents. Densitometry quantitation of the bands was performed using ImageJ software (National Institutes of Health, Bethesda, MD, USA) on a Mac OS X (Apple Computer International, Cupertino, CA, USA).

### siRNA

RNA knockdown was performed with pools of siRNA duplexes. Briefly, cells were washed and resuspended in OPTI-MEM medium, transfected with siRNA specific for human IRE1α or for human PERK and relative scrambled siRNA (Ambion), using lipofectamine RNAiMAX according to the manufacturer’s guidelines and as we previously described.^41^ After 12 h of incubation, RPMI-1640 containing 20% fetal calf serum was added without removing the transfection medium. The cells were cultured for further 60 h. After centrifugation, the medium was replaced with fresh RPMI-1640, containing 10% fetal calf serum, and the cells cultured again in the presence or not of TN or 4μ8 C + TN.

### RNA isolation and qRT-PCR

Unless otherwise indicated, reagents and equipment were purchased from Thermo Fisher Scientific. Total RNA was purified using Trizol. One μg was reverse transcribed using random primers and SuperScript II. Quantitative RT-PCR (qRT-PCR) analysis was performed using the ViiA 7 Real-Time PCR System, using the “best coverage” TaqMan gene expression assays, specific for each analyzed mRNA, according to manufacturer’s protocol. Each amplification reaction was performed in triplicate, and the average of the three threshold cycles was used to calculate the amount of transcripts in the sample (SDS 2.3 software). mRNA quantification was expressed, in arbitrary units, as the ratio of the sample quantity to the calibrator or to the mean values of control samples. All data are normalized to the mean value of three endogenous controls: GusB, β2-microglobulin and HPRT.

### Acidic compartment evaluation by LysoTracker Red

Cells were incubated for 15 min in HBSS containing LysoTracker-Red DND-99 (100 nM), a fluorescent probe with high selectivity for acidic organelles. HBSS washed cells were analyzed in a cytofluorimeter EPICS XL. Under cytofluorimetry, control and treated cells showed similar side- and forward-scatter characteristics and the gate used for each sample was the same as in control cells. The red fluorescence intensity of the gated cells was analyzed on a log scale (FL3log) and recorded as Mean Fluorescence Intensity (MFI). A minimum of 10.000 events were examined for each sample. The results are expressed according to the formula (MFI in treated cells)/(MFI in control cells)x 100 or (MFI in siRNA cells)/(MFI in scr-RNA cells)x 100.

### Statistical analysis

Results are expressed as mean ± S.D. of repeated experiment, as indicated in the figure legends. Statistical differences between the data sets were evaluated using unpaired, two-tailed Student’s *t*-test. Values of *p* < 0.05 were considered statistically significant.

## Electronic supplementary material

Supplementary Figure 1

Supplementary Figure 2

Supplementary Figure 3

Legends of supplementary figures
