# Correction to: IRE1α deficiency promotes tumor cell death and eIF2α degradation through PERK-dependent autophagy

**DOI:** 10.1038/s41420-018-0082-1

**Published:** 2018-07-26

**Authors:** Antonello Storniolo, Vincenzo Alfano, Sabino Carbotta, Elisabetta Ferretti, Livia Di Renzo

**Affiliations:** 1 Department of Experimental Medicine, Sapienza University of Rome, Rome, Italy; 2 Department of Molecular Medicine, Sapienza University of Rome, Rome, Italy; 3 Department of Surgical Sciences, Sapienza University of Rome, Rome, Italy; 4 Azienda Ospedaliera Universitaria Policlinico Umberto I, Rome, Italy

## Abstract

Following publication of the article, author Elisabetta Ferretti asked for the following institution to be added to her affiliation:

**Correction to:** *Cell Death Discovery* (2018) 4, 3 10.1038/s41420-017-0002-9 published online January 29, 2018.

Following publication of the article, author Elisabetta Ferretti asked for the following institution to be added to her affiliation:

Istituto di Ricovero e Cura a Carattere Scientifico Neuromed, Pozzilli, 86077 Isernia, Italy

So the final and correct affiliation for Elisabetta Ferretti is:

Department of Experimental Medicine, Sapienza University, 00161 Rome, Italy

Istituto di Ricovero e Cura a Carattere Scientifico Neuromed, Pozzilli, 86077 Isernia, Italy

The PDF and HTML versions of the paper have been modified accordingly.

