# Supplementary figures and images for: IRE1α deficiency promotes tumor cell death and eIF2α degradation through PERK dipendent autophagy
Source: Cell Death Discov. 2018 Jan 29;4:3. doi: 10.1038/s41420-017-0002-9 (PMC5841272; doi:10.1038/s41420-017-0002-9)

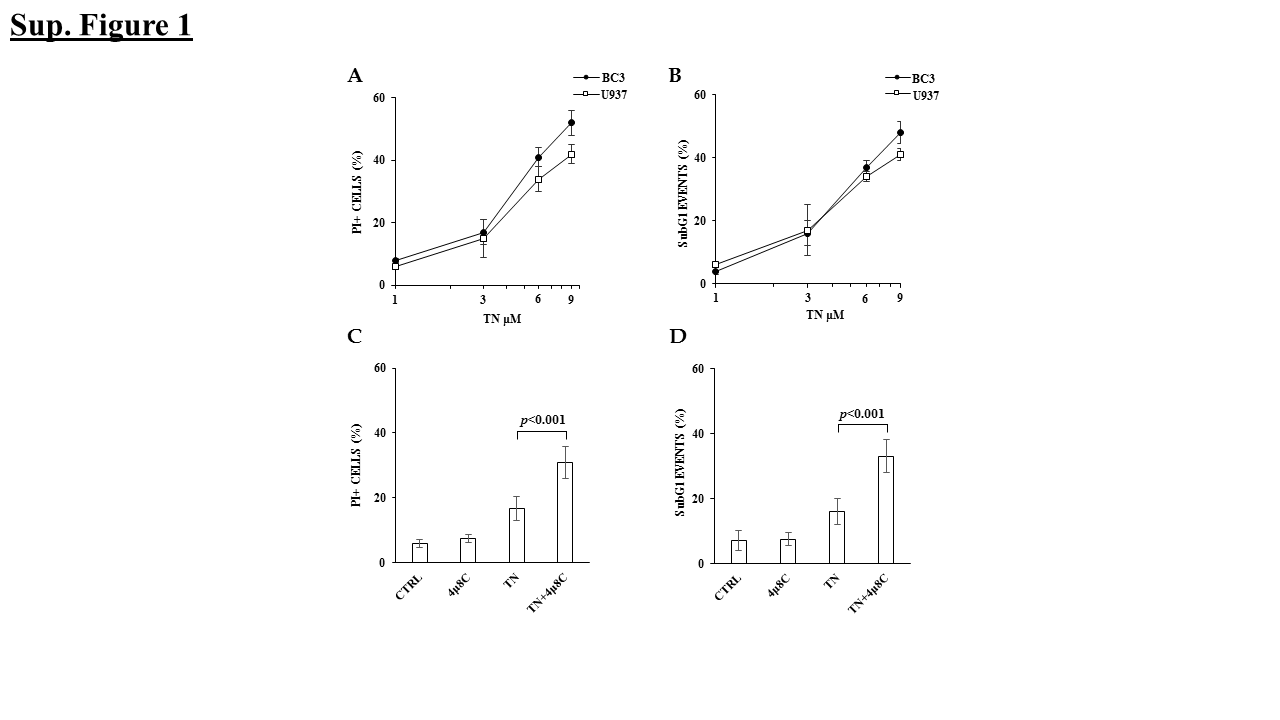

Supplement: Supplementary file 1 — Supplementary Figure 1 [file 41420_2017_2_MOESM1_ESM.tif]

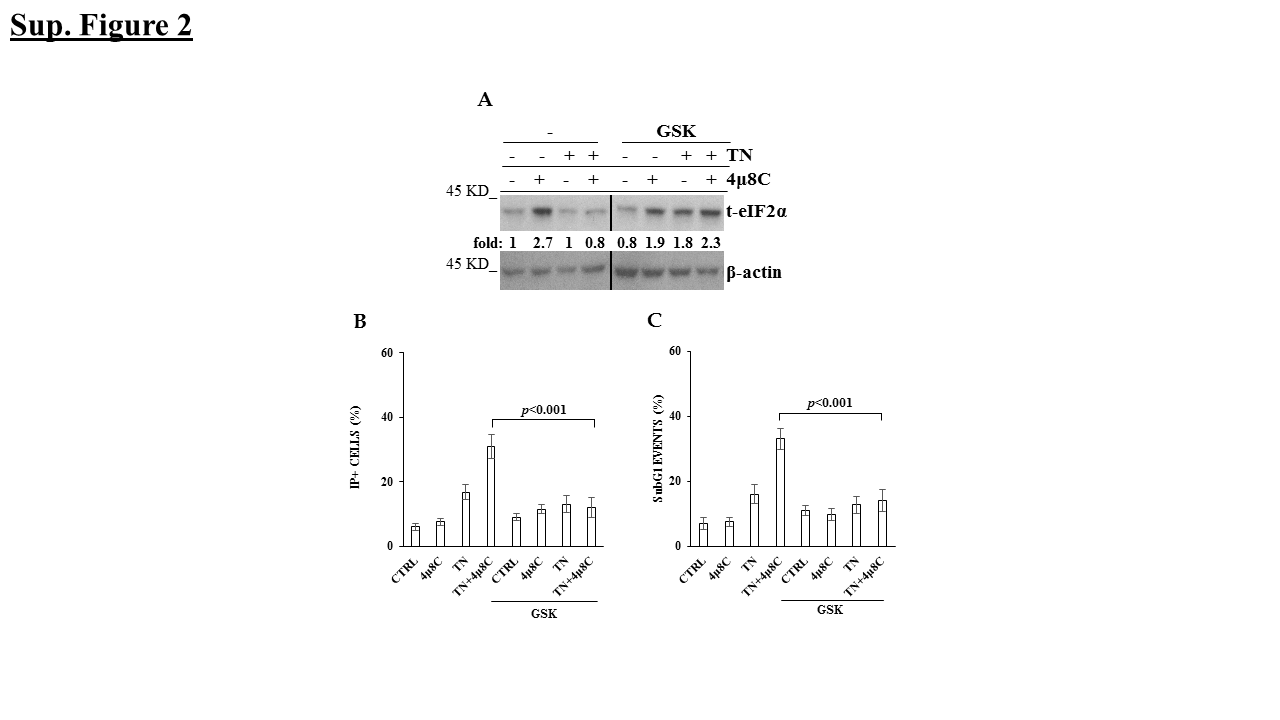

Supplement: Supplementary file 2 — Supplementary Figure 2 [file 41420_2017_2_MOESM2_ESM.tif]

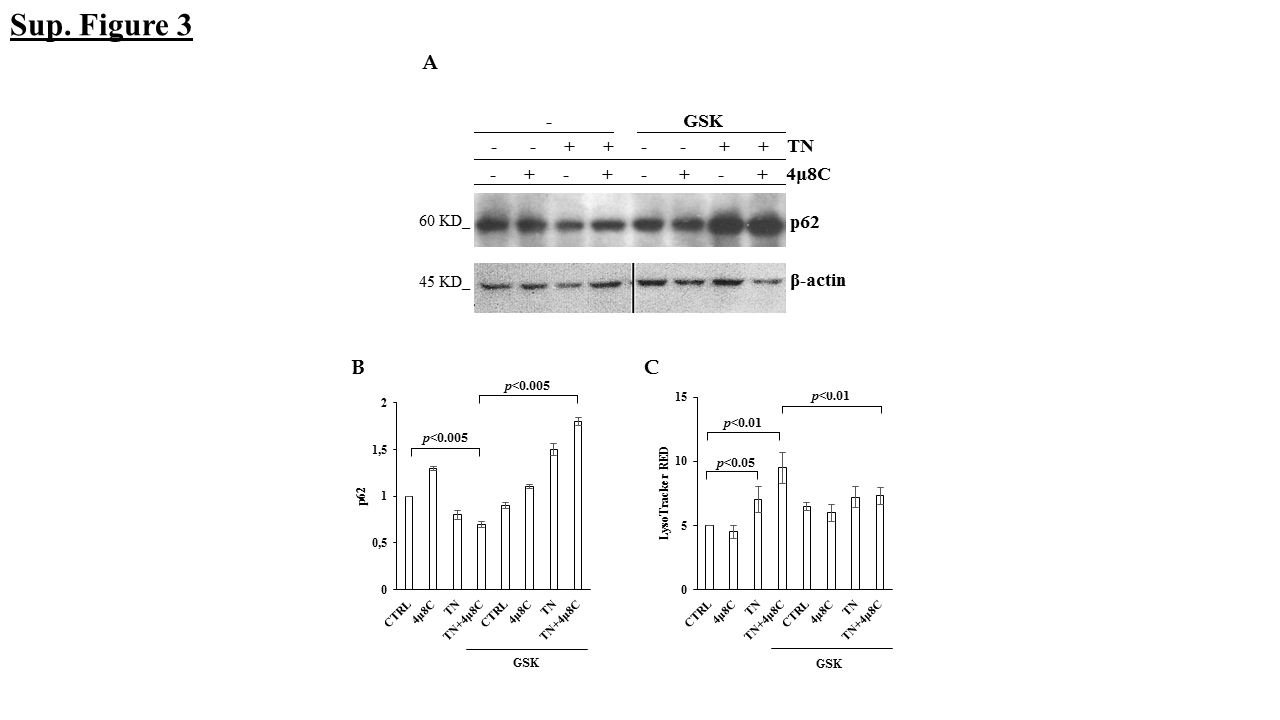

Supplement: Supplementary file 3 — Supplementary Figure 3 [file 41420_2017_2_MOESM3_ESM.tif]
