# Supplementary material for: IRE1α deficiency promotes tumor cell death and eIF2α degradation through PERK dipendent autophagy
Source: Cell Death Discov. 2018 Jan 29;4:3. doi: 10.1038/s41420-017-0002-9 (PMC5841272; doi:10.1038/s41420-017-0002-9)
Supplement: Supplementary file 4 — Legends of supplementary figures [file 41420_2017_2_MOESM4_ESM.docx]

**Legends of supplementary figures**

**Supplementary Figure 1. Cell death depends on the dose of TN and IRE1α inhibition affects the survival of BC3 cells, under ER stress.** (A,B) U937 cells and BC3 cells were treated or not with different concentrations of TN for 18 h. Cell death parameters were evaluated by calculating PI positive cells as percentage of total cells examined by cytofluorimetry (A). A portion of these cells were fixed and stained with PI and subG1 events were evaluated in the cell cycle by cytofluorimetry (B). For each parameter ≥10.000 events were acquired for each sample.

(C,D) In comparison with TN (3 μM) treatment, pretreatment with 4μ8C (12.5 μM) caused a significant increase of cell death also in BC3 cells. Data are means ± S.D. of three independent experiments. Statistical analysis by Student’s *t*-test are shown.

**Supplementary Figure 2. PERK impairment prevents eIF2α down-regulation and death of BC3 cells.** (A) Representative western blots for t-eIF2α detected in the lysates of BC3 cells pretreated or not for 30 min with GSK (10 μM) and thereafter treated or not for 18 h with TN (3 μM), 4μ8C (12.5 μM) or both drugs. eIF2α was probed with anti total eIF2α antibody, followed by peroxidase-conjugated secondary antibody. β-actin is shown at the bottom as a loading control. The values under each band were obtained using the formula: (densitometry value of the band under examination / densitometry value of the band of the corresponding β-actin) / (densitometry value of the band under examination in the lysate of untreated cells / densitometry value of the β-actin band in the lysate of untreated cells). This type of experiment was performed twice with comparable results.

Cell death parameters were evaluated in BC3 cells calculating PI positive cells as percentage of total cells examined by cytofluorimetry (B). A portion of these cells were fixed and stained with PI and subG1 events were evaluated in the cell cycle by cytofluorimetry (C). For each parameter ≥10.000 events were acquired for each sample. The reported values are means ± S.D. (N=3). Statistical analysis by Student’s *t*-test are shown.

**Supplementary Figure 3. PERK impairment prevents autophagy in ER stressed BC3 cells.** (A) Representative western blot for p62 detected in the lysates of BC3 cells pretreated or not for 30 min with GSK (10 μM) and thereafter treated or not for 18 h with TN (3 μM), 4μ8C (12.5 μM) or both drugs. p62 was probed with specific antibody, followed by peroxidase-conjugated secondary antibody. β-actin is shown at the bottom as a loading control.

(B,C) Cell death parameters were evaluated in BC3 cells calculating PI positive cells as percentage of total cells examined by cytofluorimetry (B). A portion of these cells were fixed and stained with PI and subG1 events were evaluated in the cell cycle by cytofluorimetry (C). For each parameter ≥10.000 events were acquired for each sample. The reported values are the means ± S.D. (N=3). Statistical analysis by Student’s *t*-test are shown.
